# Group singing and its effect on cortisol, alpha amylase, oxytocin, and pain threshold in patients with Parkinson's disease

**DOI:** 10.3389/fnins.2025.1569601

**Published:** 2025-05-09

**Authors:** Adiel Mallik, Tara Raessi, Arla Good, Alex Pachete, Frank A. Russo

**Affiliations:** Department of Psychology, Toronto Metropolitan University, Toronto, ON, Canada

**Keywords:** group singing, Parkinson's disease, cortisol, alpha amylase, oxytocin, pain

## Abstract

**Background:**

Parkinson's disease (PD) is a neurodegenerative disorder that causes motor deficits, including rigidity and tremors. Pain is also a common problem for people with PD that may arise from their dopamine deficit. Some patients with PD experience temporary relief from pain through group singing, which has also been shown to mitigate vocal challenges related to PD. However, no work has been conducted to elucidate the neurochemical mechanisms of action on the pain threshold. Here, we examined whether the effects of group singing on cortisol, alpha amylase, and oxytocin levels are associated with changes in pain thresholds in patients with PD.

**Methods:**

Participants with PD (*n* = 14) participated in a 12-week singing program involving weekly 45-min group singing sessions in the early afternoon. Data collection, involving saliva samples and pain thresholds, was performed pre- and post-session in the 2^nd^ (Session 1), 7^th^ (Session 2), and 12^th^ (Session 3) weeks of the program. Saliva samples were collected before and after each session by using the passive drool method. The pain threshold was assessed before and after each session by applying pressure to the finger using a dolorimeter. Saliva samples were used to assess salivary cortisol (sCORT), alpha amylase (sAA), and oxytocin (sOXT). Pain threshold, sCORT, sAA, and sOXT change scores were calculated for each session by subtracting the pre-session value from the post-session value.

**Results:**

Three mixed linear model analyses were performed to assess whether sCORT, sAA, and sOXT were associated with increased pain threshold. We found that group singing led to a significant reduction in sCORT and sAA. We also found that reductions in sCORT were significantly related to an increase in the pain threshold (*p* < 0.05). However, we did not observe any relationship between pain threshold increases and sAA or between pain threshold and sOXT.

**Conclusion:**

Group singing significantly increases the pain threshold in patients with PD, and this increase may be mediated through a reduction in cortisol levels.

## Introduction

Parkinson's disease (PD) is a progressive neurodegenerative disorder where the main symptoms are altered neural control of movement and altered control of voice, respiration, and swallowing (Stegemöller et al., 2017). Between 1990 and 2015, the number of people living with PD doubled, surpassing 6 million, and is expected to exceed 10 million by 2040 (Dorsey and Bloem, 2018). This rapid rise has led some researchers to characterize PD as a pandemic (Dorsey and Bloem, 2018; Bloem et al., 2021; Mohamed, 2024). Pain is a common but often untreated symptom of PD (Karnik et al., 2020). Group singing has been shown to improve and better manage vocal production challenges in patients with PD (Good et al., 2023). There is a growing body of evidence indicating that music can reduce pain (Hsieh et al., 2014; Guo et al., 2020; Sihvonen et al., 2022; Zhang et al., 2023; Paulander et al., 2024). Singing- and music-based interventions have been suggested as non-pharmacological treatment options for PD-related pain (Skogar and Lokk, 2016; Qureshi et al., 2021). The neurochemical mechanisms of action that have been proposed to be involved in the immediate effects of music-based activity on systemic physiology include increases in dopamine (Salimpoor et al., 2011), opioids (Mallik et al., 2017), beta-endorphins (Dunbar et al., 2012), and oxytocin (Good and Russo, 2022), and decreases in cortisol levels (Good and Russo, 2022). However, no studies have specifically examined the effectiveness of singing/music therapy in alleviating pain, specifically in patients with PD, or their neurochemical mechanisms of action (Skogar and Lokk, 2016; Qureshi et al., 2021). In this study, we investigated whether group singing can effectively alleviate pain in patients with PD and assessed the mechanisms involved in this pain reduction.

Oxytocin is a neuropeptide synthesized mainly in the paraventricular nucleus (PVN) and supraoptic nucleus (SON) of the hypothalamus (Love, 2014). Oxytocin neurons in the PVN send projections to the extra-hypothalamic regions, including the amygdala, hippocampus, nucleus accumbens, and the ventral tegmental area (VTA) (Brinton et al., 1984; Van Leeuwen et al., 1985; Fliers et al., 1986; Caffé et al., 1989; Loup et al., 1991; Krémarik et al., 1993; Ross et al., 2009). In addition to oxytocin being anatomically linked to the neurological reward system, there is evidence that oxytocin can have downstream positive effects on both dopamine and opioid systems, further reinforcing social motivation and reward (Baskerville et al., 2009; Baskerville and Douglas, 2010; Putnam and Chang, 2022). Some studies have shown that group singing increases oxytocin levels in communal choir contexts (Kreutz, 2014; Good and Russo, 2022). However, other studies have reported a decrease in oxytocin levels after singing in more competitive choir contexts (Schladt et al., 2017). Pain treatments, such as analgesics (e.g., morphine), typically target the neurological reward system in the brain. Rewards are experienced in mammals in two phases (Wise, 2004; Peciña and Smith, 2010), with distinct neurochemical correlates: an appetitive/anticipatory phase, driven by the mesotelencephalic dopaminergic pathway, and a consummatory phase, driven by both dopamine and μ-opioid receptor activation (Berridge, 2004; Wise, 2004; Berridge, 2007; Berridge and Kringelbach, 2008). Anticipatory and consummatory pleasures—wanting and liking—depend on different sites within the nucleus accumbens (NAcc). Anticipatory pleasure is linked to a widely distributed network throughout the NAcc, whereas consummatory pleasure is linked to the rostrodorsal quarter of the medial accumbens shell (Peciña, 2008). Several studies have shown that pleasurable music activates the NAcc (Menon and Levitin, 2005; Koelsch et al., 2006; Salimpoor et al., 2011). Both the NAcc and ventral tegmental area (VTA) have a high density of μ-opioid receptors (Lutz and Kieffer, 2013). Previous studies demonstrated that the opioid system mediates pleasurable responses to music (Mallik et al., 2017).

The opioid and dopaminergic systems are anatomically linked, and previous studies have shown that blocking the opioid system can reduce dopaminergic activity (Hakan and Henriksen, 1989; Spanagel et al., 1992; Benjamin et al., 1993; Taber et al., 1998; David et al., 2002; Lee et al., 2005; Pierce and Kumaresan, 2006). That is, if one pharmaceutically blocks opioid-mediated consummatory reward circuits, dopamine-mediated anticipatory reward circuits are likely to be affected simultaneously. Taken together, oxytocin is anatomically linked to both the opioid and dopaminergic systems and can positively upregulate both dopaminergic and opioid activity (Caffé et al., 1989; Baskerville et al., 2009; Baskerville and Douglas, 2010; Putnam and Chang, 2022). In turn, the dopamine and opioid systems are also anatomically linked, whereby dopamine can influence and upregulate opioid activity and vice versa (Hakan and Henriksen, 1989; Spanagel et al., 1992; Benjamin et al., 1993; Taber et al., 1998; David et al., 2002; Lee et al., 2005; Pierce and Kumaresan, 2006). This suggests that salivary oxytocin may be a useful biomarker that can be assessed in the field to determine whether the neurobiological reward system is activated in the pain mitigation process during group singing.

Parkinson's disease is characterized by the loss of dopaminergic neurons. Dopamine is a vital neurotransmitter involved in the modulation of pain perception (Blanchet and Brefel-Courbon, 2018). Indeed, it has been suggested that dopamine agonist-based pharmaceuticals, such as levodopa and rotigotine, provide a beneficial effect on nociceptive pain originating in the periphery (Trenkwalder et al., 2011; Geroin et al., 2016; Blanchet and Brefel-Courbon, 2018), whereas drugs that target the opioid system are more effective at managing central pain (Blanchet and Brefel-Courbon, 2018).

In addition to the aforementioned pain reduction mechanisms of action that largely target the central nervous system, there are also stress/pain reduction mechanisms of action that target the endocrine and hypothalamic-pituitary-adrenal (HPA) axis systems. Upon exposure to stress, the cerebral cortex signals the hypothalamus to release corticotropin-releasing hormone (CRH), which causes the pituitary gland to release adrenocorticotropic hormone (ACTH), which signals the adrenal cortex to release cortisol (Huether et al., 2017). A meta-analysis of 208 studies on acute stress under laboratory conditions reported that the maximum cortisol response was observed 20–40 min after the onset of a stressful event (Dickerson and Kemeny, 2004). Additionally, the catecholamines epinephrine and norepinephrine stimulate the alpha and beta adrenergic receptors (Huether et al., 2017), which causes the contraction of smooth muscle, including the blood vessels and beta receptors, causing an increase in heart rate, contractility, and bronchodilation (Huether et al., 2017). Music has also been shown to reduce cortisol in a natural setting as well as in stressful situations (Khalfa et al., 2003; Uedo et al., 2004; Martin et al., 2015; Helsing et al., 2016).

Salivary α-amylase (sAA) has recently emerged as a surrogate marker of acute stress associated with sympathetic activation and has been correlated with plasma epinephrine and norepinephrine levels (Nater et al., 2006; Ali and Nater, 2020). Salivary α-amylase (sAA) levels typically increase in response to physical (Chatterton et al., 1996) and psychological stresses (Rohleder et al., 2004; Yamaguchi et al., 2004; Nater et al., 2005). sAA levels also tend to increase and peak earlier than cortisol levels during the stress response process (Gordis et al., 2006; Nater et al., 2006). However, sAA levels are also affected by external factors such as sex, age, and diurnal variation (Almela et al., 2011; Ali and Nater, 2020). Although no direct studies have measured or associated sAA levels with pain in patients with PD, a recent study demonstrated that sAA could be a good indicator of mental stress in these patients (Mukaiyama et al., 2024).

In the current study, participants with PD participated in a 12-week singing program involving weekly 45-min sessions with a certified music therapist. Data collection, involving saliva samples and pain thresholds, was performed pre- and post-session in the 2^nd^ (Session 1), seventh (Session 2), and 12^th^ (Session 3) weeks of the program. Saliva samples were then assayed for oxytocin, cortisol, and α-amylase. We predicted that the participants' pain mitigation would be associated with increased oxytocin levels due to downstream increases in dopamine and endogenous opioids. However, given that patients with PD have fewer dopaminergic neurons than healthy controls, we predicted that pain mitigation would be more strongly linked to a reduction in cortisol and α-amylase levels, owing to a decrease in the HPA axis and sympathetic nervous system activity. The analyses presented in this paper constitute a secondary analysis of data from a proof-of-concept paper recently submitted to *Arts and Health*.

## Materials and methods

### Participants

The studies involving humans were approved by the Toronto Metropolitan University Research Ethics Board (REB 2018-222). All experiments were performed in accordance with the relevant guidelines and regulations. Informed consent was obtained from all the participants. We recruited 14 participants from a pre-existing community choir of people with Parkinson's disease. There were 10 males and 4 females, with a mean age of 73.8 years. There was significant participant attrition in Session 3 (35%). One additional participant joined the choir between sessions 2 and 3. Outlier removal also contributed to the reduced analyzed sample size across all sessions in all measures (Tables 1–4). This was particularly the case for salivary neurohormonal measures (sCORT, sAA, and sOXT) (Tables 2–4).

**Table 1:** Additional statistical information for comparisons of pain threshold.

**Comparison**	**Tail of test**	***P*-value**	**Effect size (Cohen's d)**	**Power**	**Degrees of freedom**	**Sample size (N)**
Session 1 Pre vs. Post	Two-tailed	0.22	0.45	0.32	12	13
Session 2 Pre vs. Post	Two-tailed	0.0036	0.63	0.43	9	10
Session 3 Pre vs. Post	Two-tailed	0.76	0.19	0.08	8	9

**Table 2:** Additional statistical information for comparisons for sCORT.

**Comparison**	**Tail of test**	***p*-value**	**Effect size (Cohen's d)**	**Power**	**Degrees of freedom**	**Sample size (N)**
Session 1 Pre vs. Post	One-tailed	0.19	0.26	0.18	8	9
Session 2 Pre vs. Post	One-tailed	0.099	0.64	0.55	8	9
Session 3 Pre vs. Post	One-tailed	0.04	1.03	0.83	7	8

### Design and procedure

The study had one within-subject factor: time (before and after singing). All singing events occurred at the same time of day (early afternoon) to minimize diurnal variations in hormones (sCORT and sAA). sCORT typically peaks upon awakening and decreases throughout the day (Matsuda et al., 2012). sCORT shows the lowest day-to-day variation upon awakening but also shows a slightly higher but similar day-to-day variation during the afternoon (Matsuda et al., 2012), and sAA typically decreases 60 min after awakening and increases thereafter over the course of the day (Nater et al., 2007). Diurnal sAA is relatively stable across time, and individual differences in the sensitivity of diurnal rhythm to environmental changes and demands (Out et al., 2013). sOXT does not appear to exhibit diurnal variations (Kagerbauer et al., 2019). Pain tolerance was assessed by applying pressure on the finger using a dolorimeter and collecting saliva using the passive drool method. Saliva samples were subsequently used to assess the salivary oxytocin, cortisol, and α-amylase levels. Data collection occurred immediately before and after a 45-min singing session. For group singing, the participants engaged in regular choir activities, including breathing exercises, warmups, and repertoire rehearsals.

### Music stimuli/group singing repertoire

The choir was directed by a music therapist (MT), and each session involved similar breathing exercises, warm-ups, and vocal exercises. There was a prioritization of ecological validity over standardization of musical repertoire to ensure participant engagement in each singing session. The MT assessed and polled participants regarding song and genre preferences to ensure that the music incorporated into exercises was known and motivational as much as possible. This occurred both formally in the form of a survey at the beginning of sessions and informally during the sessions themselves, as the group began to share their ideas with one another and the MT, after which the MT incorporated them. In this case, music spanned North American pop, rock, jazz, country, and folk from the 1950s to the 1980s, with a large focus on the 60s and 70s.

The MT also worked with music that was unknown to the participants, aiming to foster cognitive challenges or learning opportunities. However, these unknown songs or vocal exercises were repeated over all sessions, resulting in known exercises/songs as the sessions progressed. Primarily, the use of known music is vital for engagement, participation, and motivation. Further, music with a more upbeat and driving tempo and style was used for songs requiring coordination or entrainment, while more lyrical music was used for aspects of stretching and vocal elasticity.

### Pain threshold

The pain threshold was assessed using a dolorimeter, with a research assistant applying increasing pressure to the second knuckle on the palm side of the participants' index finger. Participants were instructed to indicate when the pressure became uncomfortable (by saying stop or tapping the experimenter on the hand). The pressure measured in pounds was recorded at this point. This procedure was conducted six times for each participant, alternating between the index fingers of the left and right hands to ensure consistency and to account for any potential hand-specific differences.

### Salivary hormone assays

Cortisol samples were analyzed in-house using a Salimetrics© salivary cortisol enzyme competitive immunoassay standard 96-well plate assay (ELISA) kit. The preparation and processing of the saliva samples were completed according to the commercially available instruction manual provided by the ELISA kit. Samples were tested in duplicate to control for potential pipetting errors. The average concentration values were used for the analyses. The lower limit of sensitivity of this test was 0.007 μg/dL.

The α-amylase samples were analyzed in-house using a Salimetrics© salivary α-amylase enzyme competitive immunoassay standard 96-well plate assay (ELISA) kit. Preparations and processing of the saliva samples were completed according to the commercially available instruction manual provided by the ELISA kit. The amount of α-amylase in the sample was directly proportional to the increase in the absorbance at 405 nm. A volume of 10 mL of the sample was diluted and mixed well. A volume of 8 mL of the diluted samples was pipetted into individual wells of a 96-well microtiter plate. A volume of 320 μL of preheated chromogenic substrate solution was added to each well, and the plate was rotated from 500 to 600 RPM at 37°C for 3 min. The optical density of the sample was determined at the 1-min mark and again at the 3-min mark. Samples were tested in duplicate to control for potential pipetting errors. The average concentration values were used for the analyses. The lower limit of sensitivity for this test is 0.007 μg/dL.

Oxytocin samples were frozen using dry ice and shipped to an off-site laboratory (Salimetrics©), where they were analyzed using an electrochemiluminescent assay optimized and validated for performance in saliva. Samples were tested in triplicate to control for potential pipetting error. The average concentration values were used for the analyses. The lower limit of sensitivity of this test was 8.0 pg/mL.

### Statistical methods

Data with more than 1.5 × Interquartile Range (IQR) below Q1 and more than 1.5 × IQR above Q3 were classified as outliers and removed from the analysis. Outlier removal contributed to a reduced analyzed sample size across all sessions in all measures (Tables 1–4). This was particularly the case for the salivary neurohormonal measures (sCORT, sAA, and sOXT) (Tables 1–4).

For pain threshold, sCORT, sAA, and sOXT data, pairwise permutation tests, also known as Fisher Randomization Resampling tests (FRT) (5,000 iterations), were performed to compare pre- and post-session data. Permutation tests are non-parametric and provide a good way to control the Type I error rate for multiple comparisons without making assumptions about the underlying distribution of the data (Good, 1994; Kuehl, 2000; Önder, 2007; Camargo et al., 2008).

To determine whether neurohormones (sCORT, sAA, and sOXT) were associated with changes in pain threshold, three repeated measures mixed linear model analyses were conducted using the lme4 package (Bates et al., 2015) in R. In the first model, delta pain threshold (Post-session–Pre-session) was the dependent variable, and delta sCORT (Post-session–Pre-session) and session were the predictor variables. In the second model, delta pain threshold was the dependent variable, and sAA and session were the predictor variables. In the third model, delta pain threshold was the dependent variable, and delta sOXT and session were the predictor variables.

## Results

**Figure 1:**
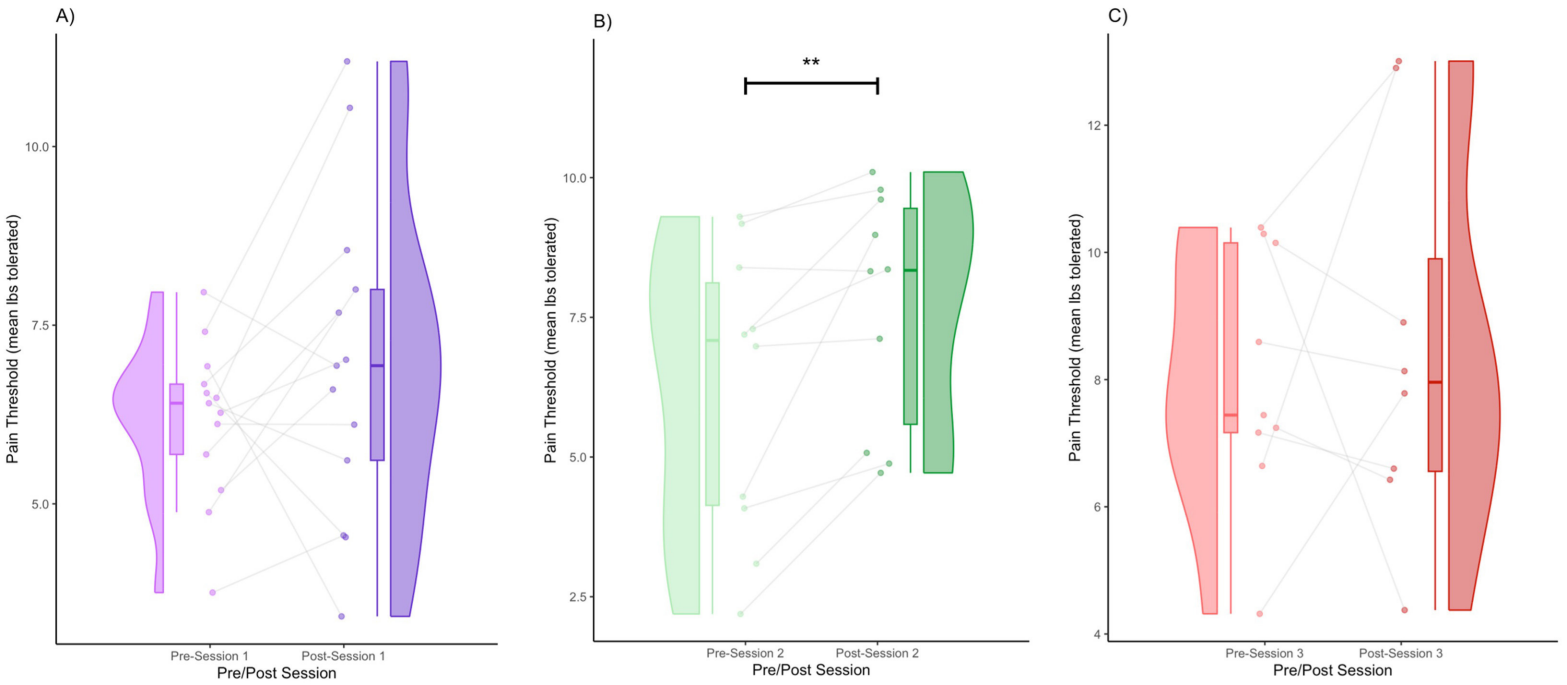
Pre- and post-session pain thresholds for Session 1 **(A)**, Session 2 **(B)**, and Session 3 **(C)** (** denotes *p* < 0.01 by FRT, 5,000 iterations).

**Figure 2:**
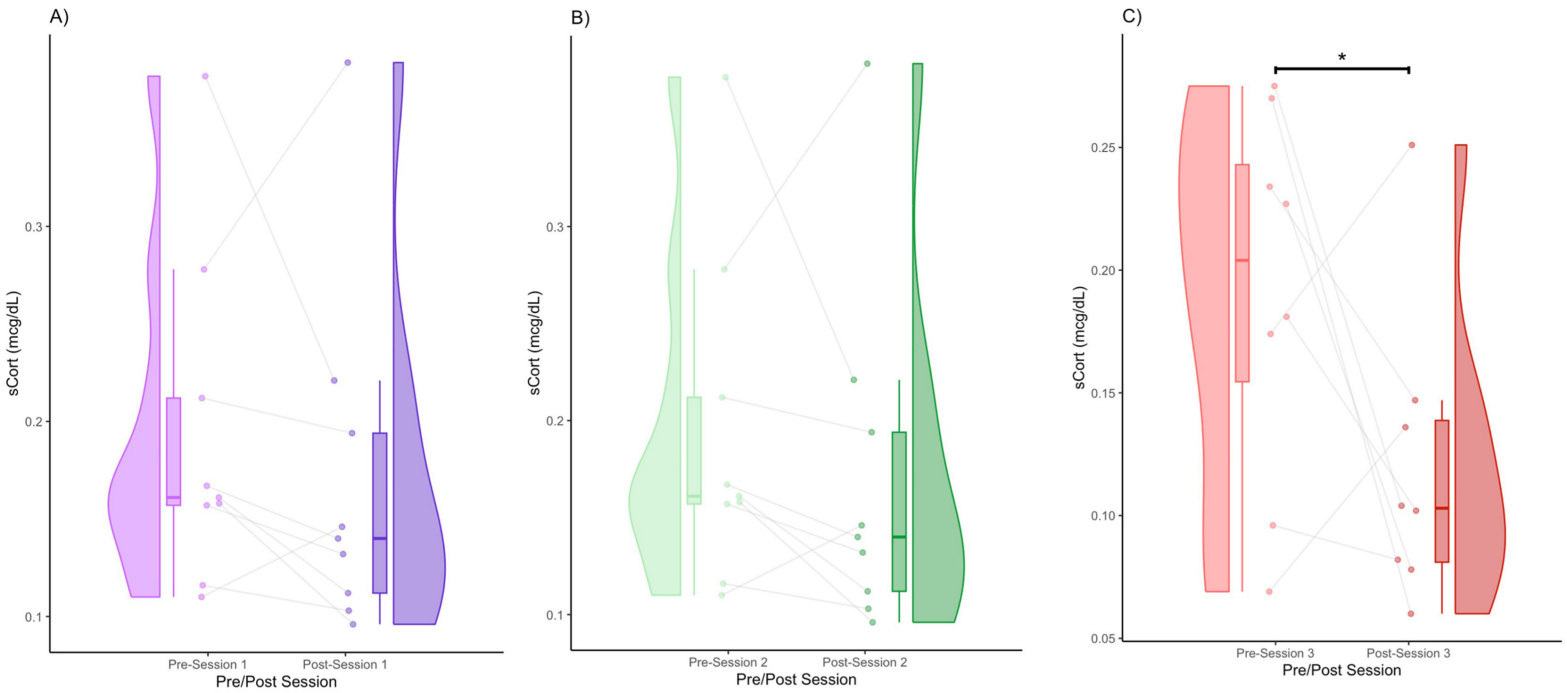
Pre- and post-session sCORT for Session 1 **(A)**, Session 2 **(B)**, and Session 3 **(C)** (* denotes *p* < 0.05 by FRT, 5,000 iterations).

**Figure 3:**
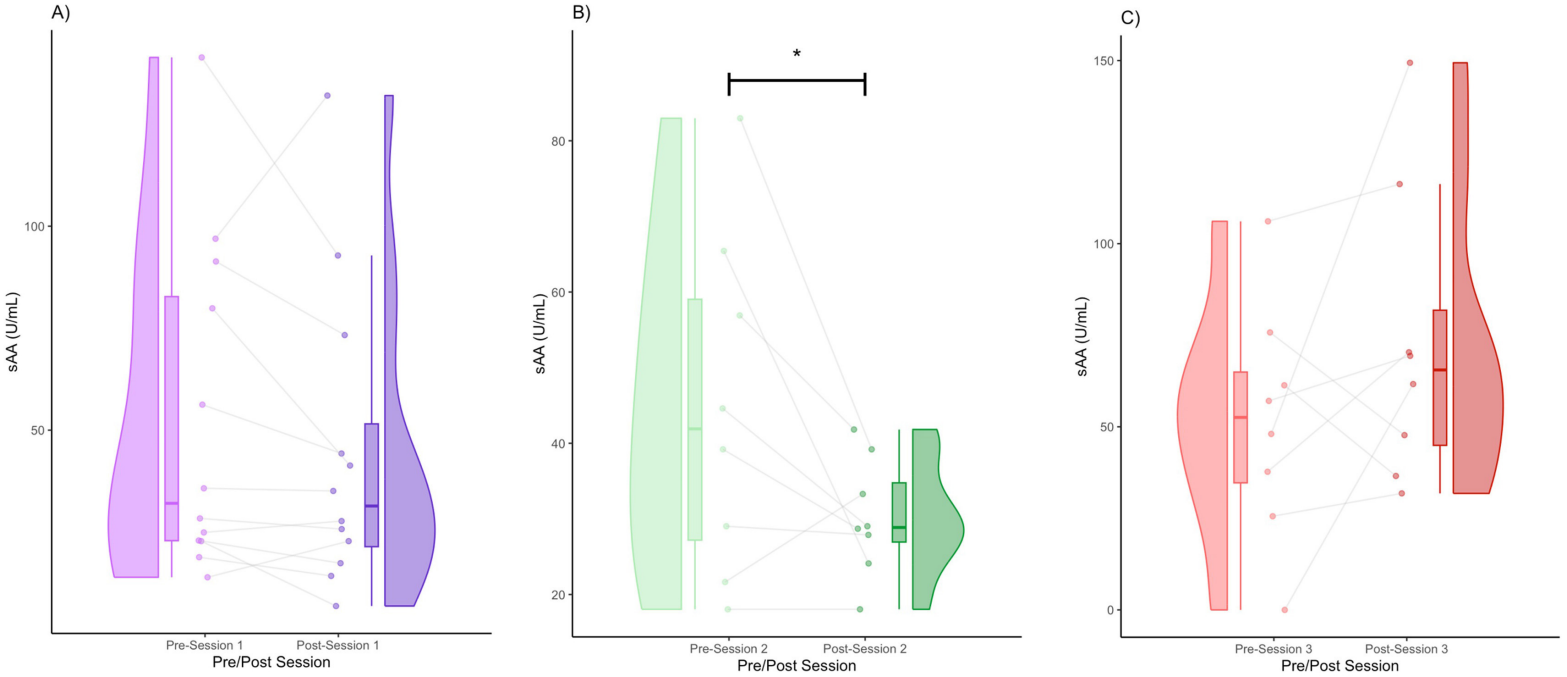
Pre- and post-session sAA for Session 1 **(A)**, Session 2 **(B)**, and Session 3 **(C)** (* denotes *p* < 0.05 by FRT, 5,000 iterations).

**Figure 4:**
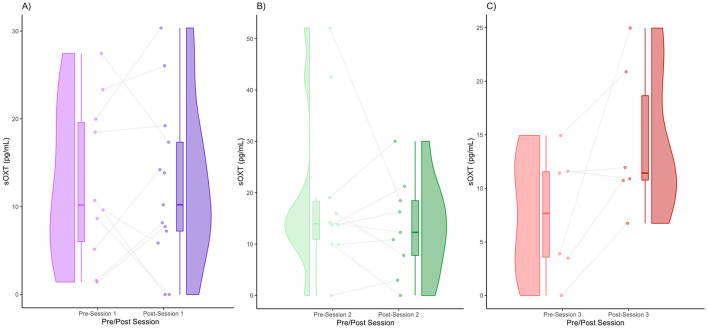
Pre- and post-session sOXT for Session 1 **(A)**, Session 2 **(B)**, and Session 3 **(C)**.

The data for pain threshold, sCORT, sAA, and sOXT are displayed in the form of raincloud plots (Figures 1–4), which consist of a violin plot indicating distribution, a boxplot displaying the central tendency and spread, and individual data points connected by lines to display within-subject changes before and after the session.

### Pain threshold

We obtained the following mean pain thresholds (± standard deviation): Session 1 (pre) = 6.18 (1.10), Session 1 (post) = 6.98 (2.27), Session 2 (pre) = 6.20 (2.58), Session 2 (post) = 7.69 (2.11), Session 3 (pre) = 8.03 (2.03), Session 3 (post) = 8.52 (3.06). In Session 1, there was a modest increase in the pain threshold from pre- to post-session. The violin plot shows a slightly broader post-session distribution (Figure 1A). Regarding within-subject patterns, some participants showed increases, whereas others remained the same or slightly decreased. Specifically, 69.2% of the participants had an increase in pain threshold, and 30.7% of participants had a decrease in pain threshold (Figure 1A). In Session 2, there was a clearer increase in the pain threshold post-session compared to Session 1. The post-session distribution shifted to the right, indicating a higher overall pain threshold (Figure 1B). Regarding within-subject patterns, the majority of participants showed an increase in the pain threshold. Specifically, 90% of the participants had an increase in pain threshold, and 10% had a decrease in pain threshold (Figure 1B). Increases in the pain threshold reached significance after Session 2 (FRT, 5,000 iterations, *p* < 0.01) (Figure 1B). In Session 3, the pain threshold increased from pre- to post-session, although the shift was somewhat less pronounced than in Session 2 (Figure 1C). The distribution was still shifted rightward post-session, but with greater variance than in Session 2 (Figure 1C). Increases were visible in many participants; however, some participants either remained the same or decreased. Specifically, 37.5% of the participants had an increase in pain threshold, and 62.5% of participants had a decrease in pain threshold (Figure 1C).

We obtained the effect sizes and power values for each statistical comparison of the pain thresholds (Table 1). In Session 2, the pre- and post-comparisons yielded a medium effect size (d = 0.63) and power value (0.43) below the ideal power of 0.80 (Table 1). Other comparisons had small effect sizes and were underpowered (Table 1).

### sCORT

We obtained the following mean sCORT concentrations (± standard deviation): Session 1 (pre) = 0.19 (0.086), Session 1 (post) = 0.17 (0.09), Session 2 (pre) = 0.16 (0.062), Session 2 (post) = 0.13 (0.047), Session 3 (pre) = 0.19 (0.076), and Session 3 (post) = 0.12 (0.06). In Session 1, there was a subtle decrease in sCORT post-session (Figure 2A). In terms of distribution, the density shifted slightly downward after the session, but with overlapping distributions (Figure 2A). In terms of within-subject patterns, many individuals showed a decrease, whereas a few showed an increase. Specifically, 77.78% of the participants had a decrease in sCORT, and 22.22% of participants had an increase in sCORT. In Session 2, similar to Session 1, there was a visually observable decrease in sCORT post-session (Figure 2B). The violin plot indicated a denser cluster of lower values with the post-session values (Figure 2B). The majority of participants showed a decrease in sCORT (77.78%), whereas some participants showed an increase in sCORT (22.22%) (Figure 2B). There was a decreasing trend in sCORT after Session 2 (FRT, 5,000 iterations, *p* < 0.1). Session 3 shows the clearest decrease in sCORT (Figure 2C). There was a marked shift toward lower post-session sCORT values in terms of distribution (Figure 2C). The majority of the participants showed a decrease in sCORT (75%), with very few showing an increase (25%) (Figure 2C). The decrease in the sCORT value in Session 3 was significant (FRT, 5,000 iterations, *p* < 0.05) (Figure 2C).

We obtained the effect sizes and power values for each statistical comparison of sCORT (Table 2). Session 2 pre- and post-comparison had a medium effect size (d = 0.55) and power value (0.83) above 0.80, making it adequately powered (Table 2). Session 3 pre-post comparison had a large effect size (d = 1.03) and power value (0.83) above 0.80, making it adequately powered. Other comparisons had small effect sizes and were underpowered (Table 2).

### sAA

We obtained the following mean sAA concentrations (± standard deviation): Session 1 (pre) = 52.78 (40.61), Session 1 (post) = 44.47 (37.04), Session 2 (pre) = 44.73 (22.56), Session 2 (post) = 30.26 (7.74), Session 3 (pre) = 51.46 (32.13), Session 3 (post) = 72.90 (40.57). In Session 1, there was a noticeable decrease in sAA levels post-session (Figure 3A). The post-session distribution was narrower and lower than the pre-session distribution (Figure 3A). The majority of participants (75%) showed a decrease in sAA levels, whereas a few participants (25%) showed an increase (Figure 3A). In Session 2, there was a strong visual decline in sAA levels post-session (Figure 3B). In terms of distribution, the post-session values clustered around a lower central tendency (Figure 3B). The majority of participants (87.5%) showed consistent sAA decreases, whereas one participant (12.5%) showed an increase (Figure 3B). sAA levels decreased significantly after Session 2 (FRT, 5,000 iterations, *p* < 0.05) (Figure 3B). In Session 3, some participants had increased sAA post-session (75%), whereas others had decreased sAA post-session (25%) (Figure 3C). The post-session violin was bimodal and wider, with increased upper-range values (Figure 3C). Individual variability was high, with several increases post-session (Figure 3C).

We obtained the effect sizes and power values for each statistical comparison of sAA (Table 3). Session 2 pre-post comparison had a large effect size (d = 0.86) and power value (0.55) below 0.80, making it underpowered (Table 3). Session 3 pre-post comparison had a medium effect size (d = 0.59) and a power value (0.30) below 0.80, making it underpowered. Other comparisons had small effect sizes and were underpowered (Table 3).

**Table 3:** Additional statistical information for comparisons for sAA.

**Comparison**	**Tail of test**	***p*-value**	**Effect size (Cohen's d)**	**Power**	**Degrees of freedom**	**Sample size (N)**
Session 1 Pre vs. Post	Two-tailed	0.22	0.21	0.10	11	12
Session 2 Pre vs. Post	Two-tailed	0.05	0.86	0.55	7	8
Session 3 Pre vs. Post	Two-tailed	0.23	0.59	0.30	7	8

### sOXT

We obtained the following mean sOXT concentrations (± standard deviation): Session 1 (pre) = 12.64 (9.16), Session 1 (post) = 12.33 (9.16), Session 2 (pre) = 19.12 (15.85), Session 2 (post) = 13.32 (9.34), Session 3 (pre) = 7.56 (5.89), and Session 3 (post) = 14.36 (6.99). In Session 1, the medians of the pre- and post-session sOXT were very similar (Figure 4A). In terms of distribution, the pre- and post-distributions were wide, suggesting inter-individual variability (Figure 4A). Some participants (60%) showed an increase in sOXT levels, whereas others (40%) showed a decrease in sOXT levels (Figure 4A). In Session 2, there appeared to be a very slight decrease in the median sOXT from pre- to post-session (Figure 4B). In terms of distribution, a few participants had higher pre-session values, which widened the pre-session distribution (Figure 4B). A total of 55.56% of the participants showed an increase in sOXT, whereas 44.45% showed a decrease in sOXT (Figure 4B). In Session 3, there was a subtle increase in the median oxytocin level post-session (Figure 4C). There was a slight upward shift and tightening of the post-session values (Figure 4C). Some individuals (83.33%) showed increased post-session sOXT, whereas one participant (16.66%) showed decreased sOXT (Figure 4C). There were no significant differences in the sOXT after any of the sessions (Figures 4A–C).

We obtained the effect sizes and power values for each statistical comparison of sAA (Table 4). Session 2 pre-post comparison had a small-to-medium effect size (d = 0.45) and power value (0.24) below 0.80, making it underpowered (Table 4). Session 3 pre-post comparison had a large effect size (d = 1.05) and a power value (0.55) below 0.80, making it underpowered. Other comparisons had small effect sizes and were underpowered (Table 4).

**Table 4:** Additional statistical information for comparisons for sOXT.

**Comparison**	**Tail of test**	***p-*value**	**Effect size (Cohen's d)**	**Power**	**Degrees of freedom**	**Sample size (N)**
Session 1 Pre vs. Post	Two-tailed	0.93	0.03	0.05	12	13
Session 2 Pre vs. Post	Two-tailed	0.36	0.45	0.24	9	10
Session 3 Pre vs. Post	Two-tailed	0.11	1.05	0.55	5	6

### Relationship between delta sCORT and pain threshold

**Figure 5:**
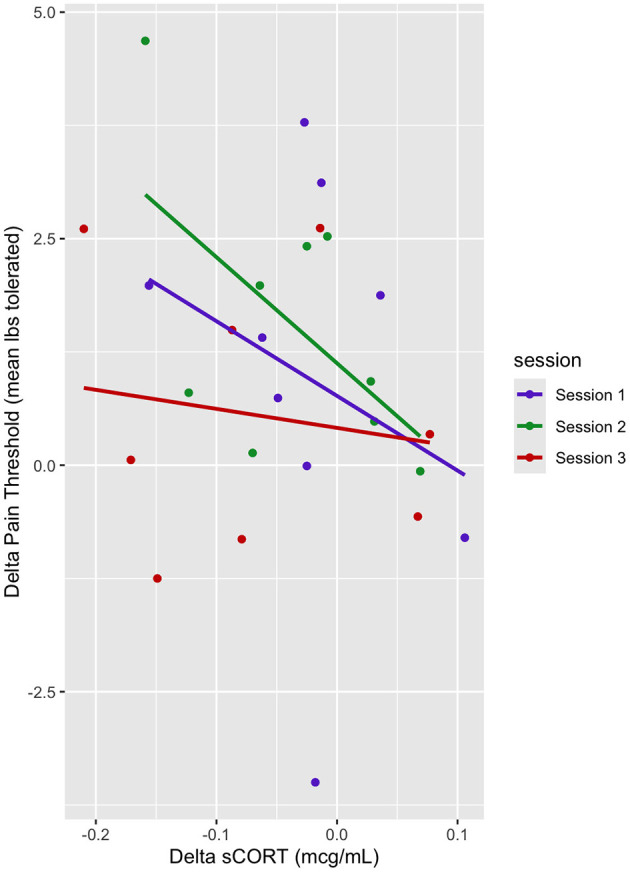
Dot plot of delta pain threshold vs. delta sCORT. Session trendlines are generated using a generalized linear model.

In this mixed linear model with delta pain threshold as the dependent variable and delta cortisol and session (sessions 1–3) as predictor variables, we found a significant interaction term of delta sCORT and session on delta pain thresholds (b = 16.65, t = 2.23, *p* < 0.05). Decomposition of the simple slopes revealed a significant relationship between delta sCORT and delta pain threshold for Session 1 (b = −16.65, t = −2.23, *p* < 0.05) and Session 2 (b = −21.97, t = −3.12, *p* < 0.01); however, there was no significant relationship for Session 3 (b = −5.31, t = −0.58, *p* > 0.05). This suggests that for sessions 1 and 2, there was a significant relationship between delta sCORT and the pain threshold, where for every unit of increase in delta pain threshold, there was a decrease in delta sCORT (Table 5, Figure 5).

**Table 5:** Delta sCORT and delta pain threshold linear mixed model fixed effects.

	**Estimate**	**Std. Error**	**Df**	**T value**	**Pr(>|t|)**
Delta sCORT	−7.14	2.86	47.26	−2.50	0.016
Session 2	0.49	0.50	45.62	0.98	0.33
Session 3	−0.69	0.54	47.69	−1.26	0.21

### Relationship between delta sAA and pain threshold

**Figure 6:**
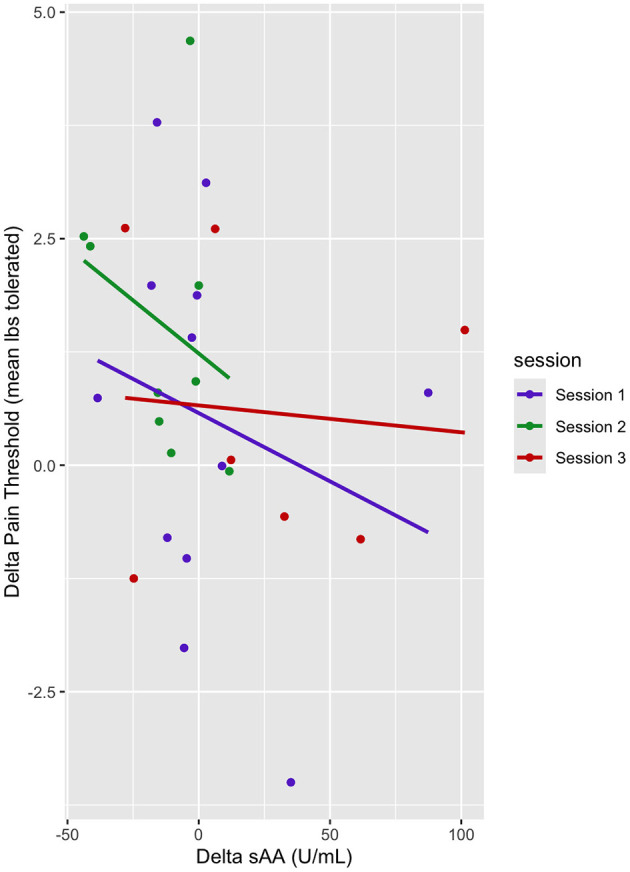
Dot plot of delta pain threshold vs. delta sAA. Session trendlines are generated using a generalized linear model.

In this mixed linear model, delta pain threshold was the dependent variable, and delta sAA and sessions (sessions 1–3) were predictor variables. There was no main effect of delta sAA on the pain threshold (b = −0.019, t = −2.06, *p* > 0.05) (Table 6, Figure 6). There was a marginal effect of Session 2 on the delta pain threshold (b = 0.93, t = 1.88, *p* < 0.1) (Table 6, Figure 6). There was no interaction between the effect of delta sAA and Session 3 on the delta pain threshold (b = 0.025, t = 1.74, *p* > 0.05) (Figure 6).

**Table 6:** Delta sAA and delta pain threshold linear mixed model fixed effects.

	**Estimate**	**Std. Error**	**Df**	**T value**	**Pr(>|t|)**
Delta sAA	−0.0038	0.0076	51.99	−0.50	0.62
Session 2	0.93	0.49	47.53	1.88	0.066
Session 3	0.12	0.54	46.79	0.23	0.82

### sOXT and pain threshold

**Figure 7:**
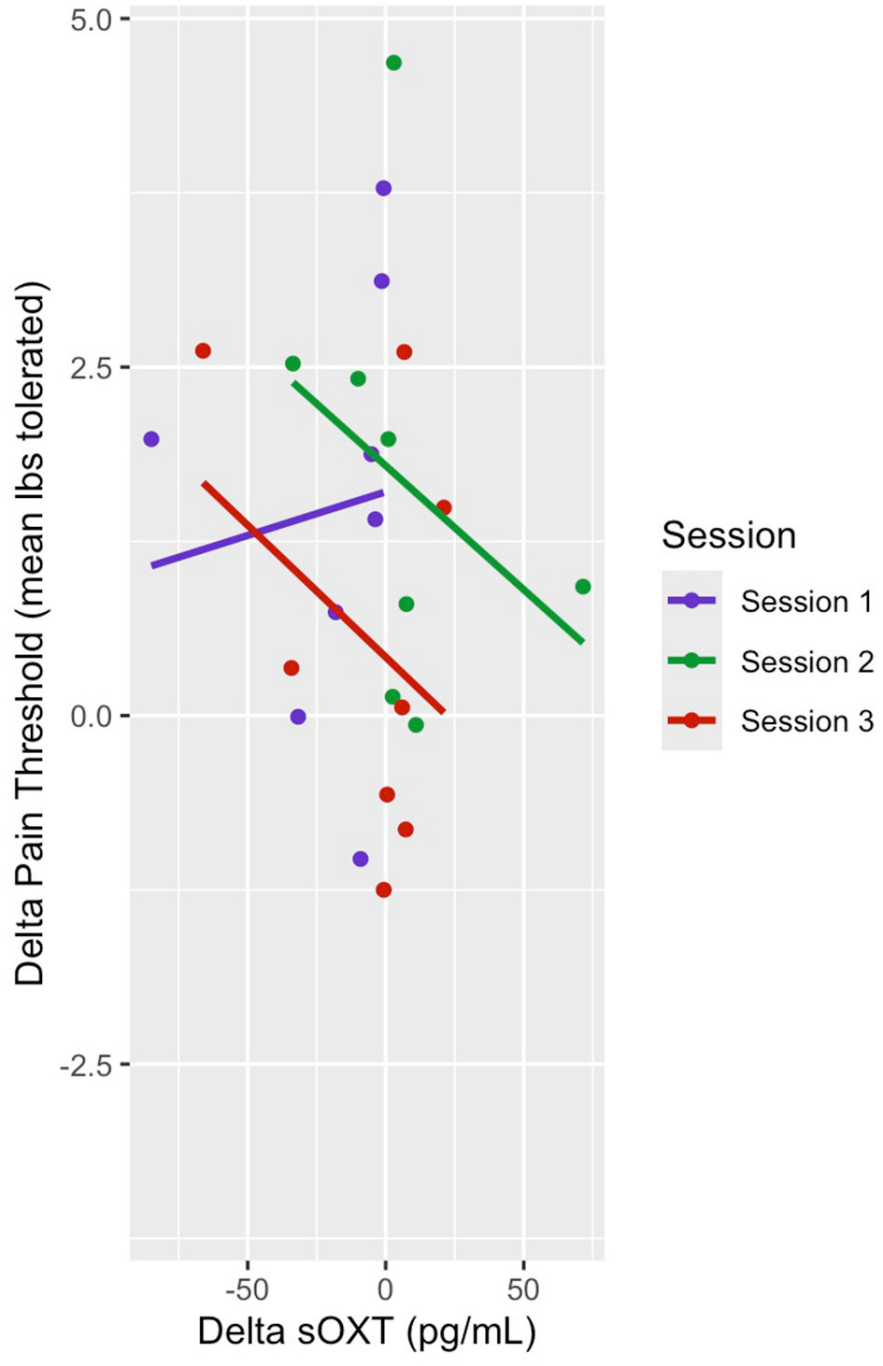
Dot plot of delta pain threshold vs. delta sOXT. Session trendlines are generated using a generalized linear model.

In this mixed linear model, the pain threshold was the dependent variable, and sOXT, session (sessions 1–3), and pre- and post-sessions were the predictor variables. There was no main effect of sOXT on the pain threshold, and there was no main effect of the session on pain threshold (Table 7). There was no interaction effect of delta OXT and Session 3 on the pain threshold (b = −0.01, t = −0.49, *p* > 0.05) (Figure 7).

**Table 7:** Delta OXT and delta pain threshold linear mixed model fixed effects.

	**Estimate**	**Std. Error**	**Df**	**T value**	**Pr(>|t|)**
Delta sOXT	−0.01	0.0086	10.98	−1.71	0.12
Session 2	0.021	0.57	8.88	0.038	0.97
Session 3	1.10	0.56	9.58	1.99	0.08

## Discussion

This study aimed to investigate the neurohormonal effects of group singing and its association with changes in pain thresholds in patients with Parkinson's disease. We found a general trend of increasing pain thresholds, with the trend turning significant after Session 2 (Figure 1). This validates previous work, which showed that music performance increases the pain threshold (Dunbar et al., 2012). We also found a general trend toward decreased cortisol levels; however, it only reached significance in Session 3. This reduction in cortisol is consistent with several prior studies showing that singing reduces cortisol levels (Sakano et al., 2014; Bowling et al., 2022; Good and Russo, 2022). We also found significant decreases in alpha amylase, which also validates previous work showing a decrease in alpha amylase after singing (Sanal and Gorsev, 2014). Interestingly, in the context of high-stress settings, such as those that may be associated with competitive choirs, performance anxiety often results in pre-post increases in alpha amylase and cortisol levels (Beck et al., 2000; Fancourt et al., 2015; Turan et al., 2022). We also found that a decrease in cortisol levels was associated with an increase in the pain threshold. This finding is consistent with a large body of work outside the context of music-based interventions, which indicated that increases in cortisol are associated with increases in pain sensitivity (Choi et al., 2012; Benson et al., 2019). Similarly, increases in salivary alpha amylase have been found to correlate with increases in subjective heat pain perception (Wittwer et al., 2016) and were positively correlated with increases in subjective pain in patients suffering from chronic pain (Shirasaki et al., 2007). Unlike previous studies, we did not find a significant relationship between alpha amylase levels and the pain threshold. This may be because of the reduced statistical power resulting from a small sample size (Table 3).

We found no significant changes in oxytocin pre- to post-session and no significant association between oxytocin and increased pain tolerance. This lack of change may have been due to the dopamine agonist medications of PD participants, which have been shown to interfere with oxytocin signaling (Blanchet and Brefel-Courbon, 2018). We excluded seven outlier participants with oxytocin concentrations far above the normal range, which may have been due to medication-related effects. Furthermore, it is possible that this choir may not have been long enough to lead to a significant increase in oxytocin levels because oxytocin is often associated with social bonding and trust. Previous studies have indicated that oxytocin level increases are more likely to occur after the groups are well established (Good and Russo, 2022; Good et al., 2023).

Regarding stress reduction mechanisms, in patients with PD, the most active stress reduction pathways appear to be centered around the HPA axis and Autonomic Nervous System (ANS), which contain the downstream salivary biomarkers of cortisol and alpha amylase, respectively.

Cortisol can influence pain threshold through four pathways. First, cortisol influences the β-endorphin levels during stress. Specifically, an acute decrease in cortisol can increase opioid sensitivity, pain inhibition, and pain threshold, which is observed in individuals with Addison's disease who have low cortisol levels but display heightened opioid responses to pain (Higginbotham et al., 2022; Lubejko et al., 2022; Shenoy and Lui, 2023). Second, chronic stress and high cortisol levels sensitize pain pathways by increasing N-methyl-D-aspartate (NMDA) receptor activation and reducing gamma-aminobutyric acid (GABA)ergic inhibition in the dorsal horn. A cortisol decrease can reverse these effects, as stress-related hyperalgesia in some cases is alleviated by a reduction in cortisol (Hannibal and Bishop, 2014; Trevino et al., 2022). Third, cortisol inhibits dopamine release in the mesolimbic system, which affects pain modulation and reward processing (Hannibal and Bishop, 2014; Higginbotham et al., 2022; Lubejko et al., 2022). A decrease in cortisol levels reduces this inhibition, thereby increasing dopamine activity (Hannibal and Bishop, 2014; Higginbotham et al., 2022; Lubejko et al., 2022). Cortisol reduction leads to increased noradrenaline levels in the locus coeruleus, which enhances descending pain inhibition, a process by which the brain sends signals to the spinal cord to block pain before conscious perception (Hannibal and Bishop, 2014; Higginbotham et al., 2022; Lubejko et al., 2022). Fourth, although cortisol is anti-inflammatory, chronic exposure can lead to glucocorticoid receptor downregulation, thereby reducing its effectiveness (Hannibal and Bishop, 2014). An acute decrease in cortisol levels may temporarily reset inflammation-reducing neuroinflammation that is linked to pain (Hannibal and Bishop, 2014).

It is important to note that there is some debate as to whether salivary alpha-amylase may represent purely sympathetic or parasympathetic activity, or a combination of both (Nater and Rohleder, 2009; Warren et al., 2017). This seems to be dependent on the research context, specifically whether acute stress or pharmacological challenges were applied to the participants (Ali and Nater, 2020). However, alpha amylase can be broadly used as a biomarker to determine ANS functioning in the context of behavioral medicine (Ali and Nater, 2020). Since acute stress or pharmacological challenge was not a part of the study design, alpha amylase likely represents ANS functioning in the context of this study. The pre- and post-session trends in alpha amylase levels were not consistent across all three sessions. There was a significant decrease in the sAA levels after Session 2 (Figure 3B). However, there were no significant pre-post changes in sAA levels in sessions 1 and 3 (Figures 3A, C). We speculate that this may be because of three main reasons. First, we had fewer participants who returned to Session 3 and one new participant who returned to Session 3. This newcomer may have had more stress since he/she did not participate in many of the prior choir sessions, as the participants did in sessions 1 and 2. The second reason may be that ANS dysregulation typically occurs in chronic stress-related pathologies, such as PD, which may affect the results that we observed in alpha amylase here (Ali and Nater, 2020). Third, the sample size of this study was small and lacking in statistical power (Table 3).

This study has several limitations; thus, the results should be interpreted with caution. First, the small sample size and missing data because of attrition and outlier removal led to reduced statistical power, which may explain the lack of consistency in obtaining statistically significant results across all sessions. Additionally, there were fewer repeat participants that came in for session 3, and one participant was a new participant who had no prior experience with the choir. Another limitation was the lack of a sufficiently sized equivalent age-matched healthy aging control group to see how healthy older adults are impacted by group singing compared to those with PD, as well as the lack of an active control group with PD performing a non-musical activity to help address any potential confounds. Other limitations reflect a lack of standardization across choirs. In order to enhance the potential for knowledge translation, a choice was made to select a musical repertoire with input from choir participants. While this choice had the benefit of enhancing participant engagement and ecological validity, it came at the expense of standardization of duration, intensity, and types of activity performed in each singing session. Future studies should aim to standardize the duration and intensity to reduce the variability in each singing session and determine whether more consistent results can be obtained across sessions.

A final limitation of this study is the use of dolorimeters to assess pain tolerance. We cannot rule out the possibility that demand characteristics may have affected pain thresholds in some manner. For example, participants may attempt to tolerate more pain in the presence of a research assistant than they would otherwise (Tousignant, 2010). In future work, we will consider the use of additional pain scales (e.g., visual analog scales) to complement the dolorimeter to try and limit these demand characteristics. However, given the limitations of logistics, time, and special populations, pain testing with a dolorimeter or something similar (e.g., blood pressure cuff) appears to be the optimal method for assessing pain tolerance.

We also had only one neurohormonal output, in this case, salivary oxytocin, which represented the central nervous system's response to pain. Although oxytocin plays an important role in social bonding, it is downstream of other key biomarkers, such as beta-endorphin, which is typically more involved in neurohormonal responses to pain and stress. Future studies should include salivary beta-endorphin as a measure.

Future studies should also include one or two follow-up sessions or perhaps additional data collection sessions later in the day to determine how long the effects of group singing interventions last both in the short term (intra-day) and the medium to long term (1–6 months) after the intervention. Prior work on healthy adults suggests that the beneficial effects on pain perception and mood of a group singing intervention may last up to 6 months after the intervention (Kenny and Faunce, 2004; Galinha et al., 2023).

## Conclusion

In this study, we found that group singing significantly increased the pain threshold in patients with PD and that significant reductions in cortisol were associated with this increase in pain threshold. We also found no significant changes in oxytocin or any relationship between oxytocin and the pain threshold. To our knowledge, this is the first study examining how group singing influences cortisol, alpha amylase, and oxytocin in patients with PD and how changes in these neurohormones may be associated with increases in pain threshold. We hope this provides the impetus for future studies to explore how group singing affects neurohormones and how these changes may, in turn, influence health outcomes in other populations that suffer from chronic disease.

## Data Availability

The raw data supporting the conclusions of this article will be made available by the authors, without undue reservation.
